# Adrenocortical Carcinoma: An Exceptional Survival Span Across a Tailored Management

**DOI:** 10.3390/diagnostics16183006

**Published:** 2026-09-17

**Authors:** Mara Carsote, Oana-Claudia Sima, Mihai Costachescu, Dana Terzea, Ana-Maria Gheorghe, Anda Dumitrascu, Teodor Ionut Constantin, Claudiu Nistor, Augustin Dima

**Affiliations:** 1 Department of Endocrinology, Carol Davila University of Medicine and Pharmacy, 020021 Bucharest, Romania; carsote_m@hotmail.com; 2 Department of Clinical Endocrinology V, “C.I. Parhon” National Institute of Endocrinology, 011863 Bucharest, Romania; oana-claudia.sima@drd.umfcd.ro (O.-C.S.); ana-maria.gheorghe@drd.umfcd.ro (A.-M.G.); 3 PhD School, Carol Davila University of Medicine and Pharmacy, 020021 Bucharest, Romania; 4 Department of Radiology and Medical Imaging, “Dr. Carol Davila” Central Military University Emergency Hospital, 010825 Bucharest, Romania; 5 Department of Pathology, “C.I. Parhon” National Institute of Endocrinology, 011863 Bucharest, Romania; 6 Oncoteam Diagnostics, 010719 Bucharest, Romania; 7 Department of Radiology and Medical Imaging, “C.I. Parhon” National Institute of Endocrinology, 011863 Bucharest, Romania; anda.dumitrascu@gmail.com; 8 Oncology Outpatient Compartment, “C.I. Parhon” National Institute of Endocrinology, 011863 Bucharest, Romania; teodorc.med@gmail.com; 9 Department 4—Cardio-Thoracic Pathology, Thoracic Surgery II Discipline, “Carol Davila” University of Medicine and Pharmacy, 020021 Bucharest, Romania; claudiu.nistor@umfcd.ro; 10 Thoracic Surgery Department, “Dr. Carol Davila” Central Emergency University Military Hospital, 010825 Bucharest, Romania; 11 Department of Surgery, “Dr. Carol Davila” Central Military University Emergency Hospital, 010825 Bucharest, Romania; dimagusti@gmail.com

**Keywords:** adrenalectomy, adrenal, mitotane, adrenal insufficiency, ACTH, lung metastasis, computed tomography, levothyroxine

## Abstract

Adrenocortical carcinoma (ACC) represents a very rare (an incidence of 1–2 cases per million) and aggressive malignancy (an overall 5-year survival of 15%). Less than 15–20% of cases involve a single metastatic disease. In this presentation, we documented a most exceptional survival span in a case of ACC, between the female patient’s age of 50 and the current age of 66. The subject underwent an open right adrenalectomy three times (at first diagnosis, after recurrence 2 years later, and, most recently, 15 years after the initial diagnosis amid a multi-visceral resection). She initially displayed Cushing syndrome, which remitted post-surgery. While the first pathological report established an adrenocortical adenoma, after relapse, low-grade ACC was confirmed and the first tumor mass was re-assessed as ACC based on a similar pathological profile. Following the second surgery and ACC recognition, she started mitotane therapy for 5 years (which she tolerated well, although she developed primary adrenal insufficiency). During this period, a single metastatic disease was detected at the pulmonary level, and she underwent a successful thoracic surgical resection of a 2.5 cm metastasis in the left inferior lobe (2 years after the second adrenalectomy). After this 5-year course of mitotane, the disease was stable, and a 4-year withdrawal period was followed by a relapse with local adrenal recurrence and growth of several lung nodules. Mitotane was re-started, and the en bloc resection included the right adrenal tumor (of 12 cm), right kidney, three liver segments, retro-hepatic inferior vena cava, and (partial) left renal vein. The reconstruction of the inferior vena cava required a Dacron graft of 12 cm in addition to a synthetic graft, which was placed between the left renal vein and the first graft. During surgery and in the following weeks, she presented with several episodes of acute adrenal insufficiency despite continued prompt replacement. A peak ACTH of 369 (Normal: 7.2–63.3) pg/mL was reached at one point. Renal function remained stable. In the meantime, she continued levothyroxine replacement (50 to 75 µg/day) for mitotane-induced primary hypothyroidism. Postoperative, low-grade ACC was confirmed with a Ki67 of 15%, as previously found. She re-started mitotane in month 3 and completely recovered following 9 months, while an abdominal CT scan confirmed no adrenal remnants. Further close surveillance was mandatory. A particular aspect involves the patient’s options (personalized management), as she declined chemotherapy, immunotherapy or radiotherapy at any point in life and delayed the third adrenalectomy during the COVID-19 pandemic and early post-pandemic years. This case highlights an exceptional survival span across two decades with an ailment with a generally poor prognosis. Another particular aspect involves a complex multi-segmental resection, which requires a highly skilled surgical team; only a limited number of cases have previously been published, and, to the best of our knowledge, there is no previous data with respect to a third intervention after 15 years of surviving with ACC.

**Figure 1:**
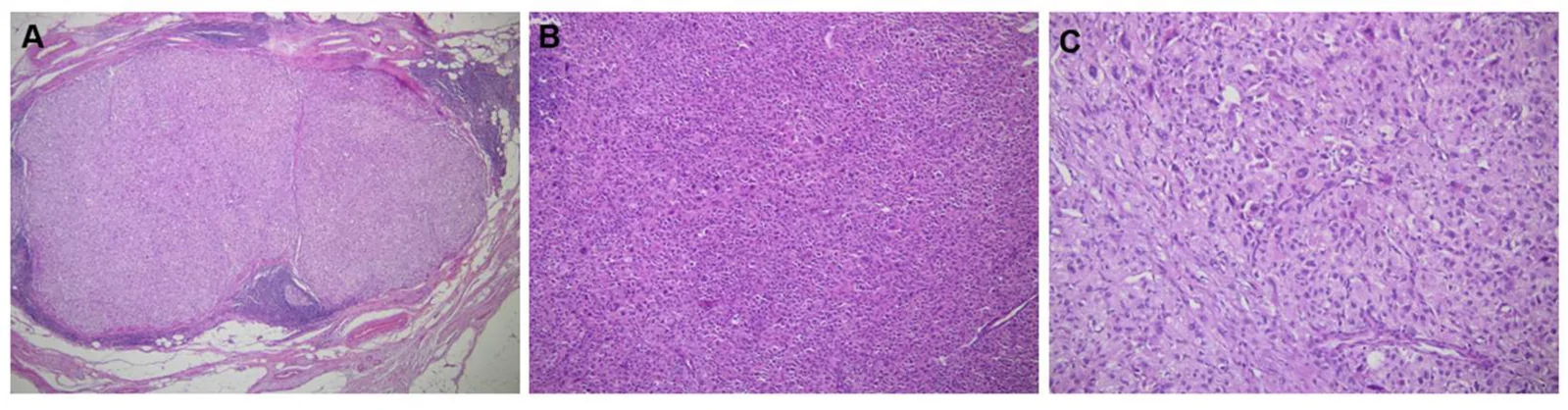
This is a 66-year-old female who was diagnosed with (adrenal) Cushing’s syndrome at the age of 50 (in 2009). She underwent an uneventful right open adrenalectomy of a 12 cm adrenal tumor. The (first and second) postoperative histological reports were consistent with an adrenocortical adenoma. Two years later, a right adrenal tumor mass of 3.6 cm was detected in the same area, and a re-do (open) adrenalectomy was performed. This time, the tumor was confirmed as being a low-grade adrenocortical carcinoma (ACC) with a Weiss score of 3, and a revision of the pathological analysis for the initial tumor showed the same features. [Histological report in 2009 (hematoxylin-eosin): (**A**) magnification 4X; (**B**) 10X; (**C**) 20X].

**Figure 2:**
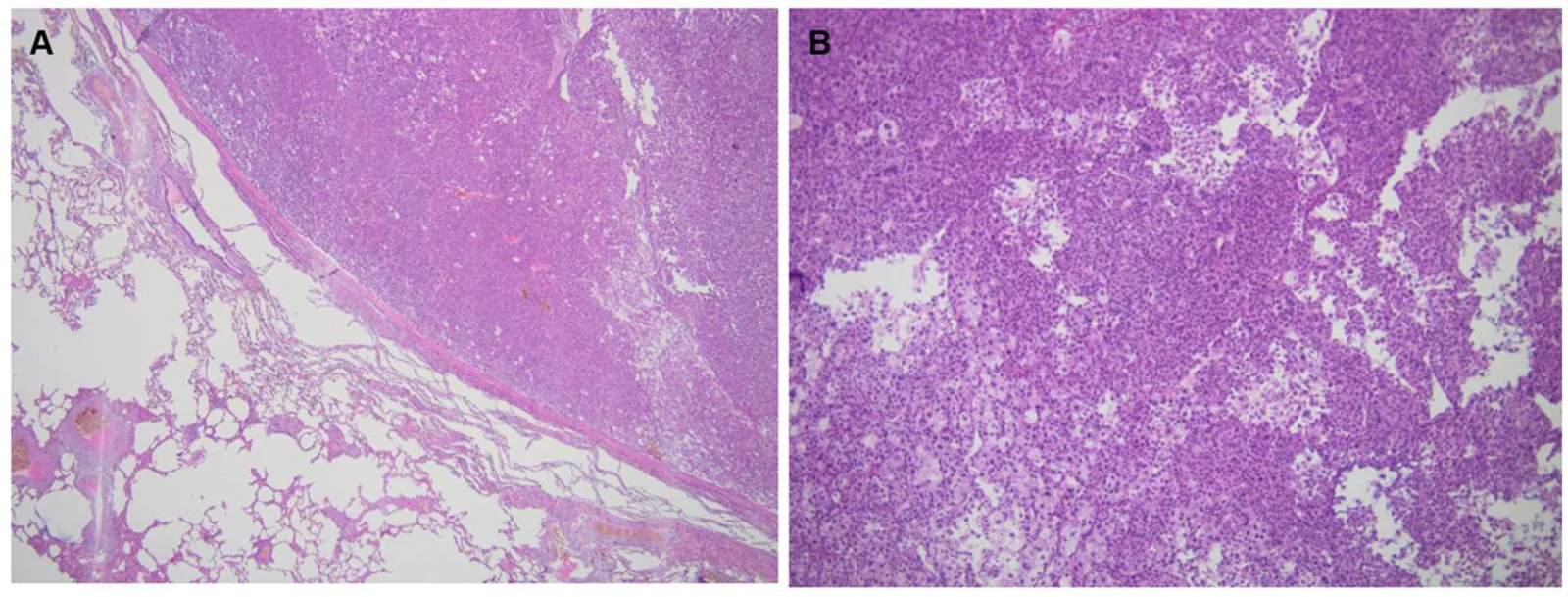
The patient started oral mitotane therapy in 2012 (Figure S1) and developed chronic primary adrenal insufficiency requiring daily oral glucocorticoid and mineralocorticoid replacement. Two years later (at the age of 54, in 2014), she was found to have a single metastatic disease at the pulmonary level (without synchronous adrenal recurrence). She refused chemotherapy and underwent the resection of the left inferior pulmonary lobe. A 2.5 cm metastasis was confirmed as originating from ACC with a similar pathological profile [Histological report in 2011 (hematoxylin-eosin): (**A**) magnification 4X; (**B**) 10X].

**Figure 3:**
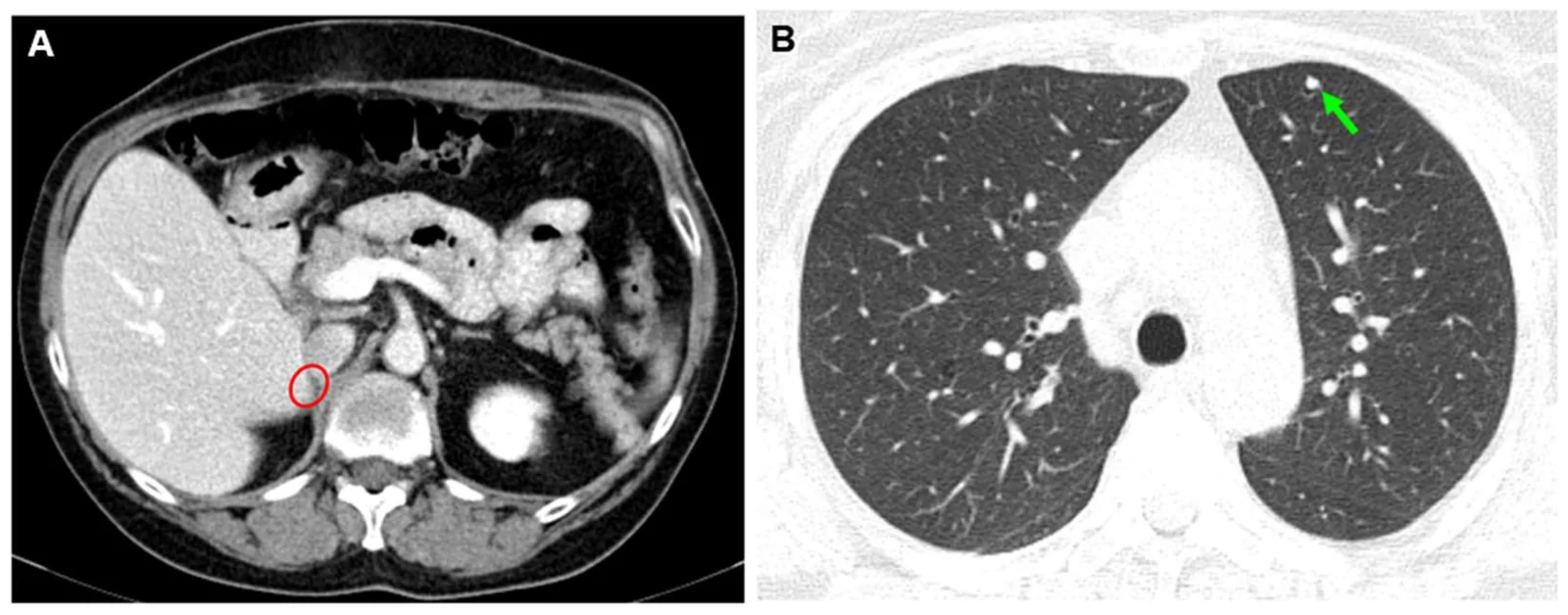
Within the year following thoracic surgery, the computed tomography (CT) showed no local recurrence at the pulmonary and abdominal intervention sites [(**A**) postsurgical fibrosis (red circle) at the place of right adrenalectomy (contrast-enhanced CT scan, axial plane, venous phase)]. In addition, a novel pulmonary micronodule of 4.7 mm in the anterior segment of the left upper lobe was detected on CT scan [(**B**) left upper lobe with micronodule (green arrow) at contrast-enhanced CT scan, axial plane, lung window].

**Figure 4:**
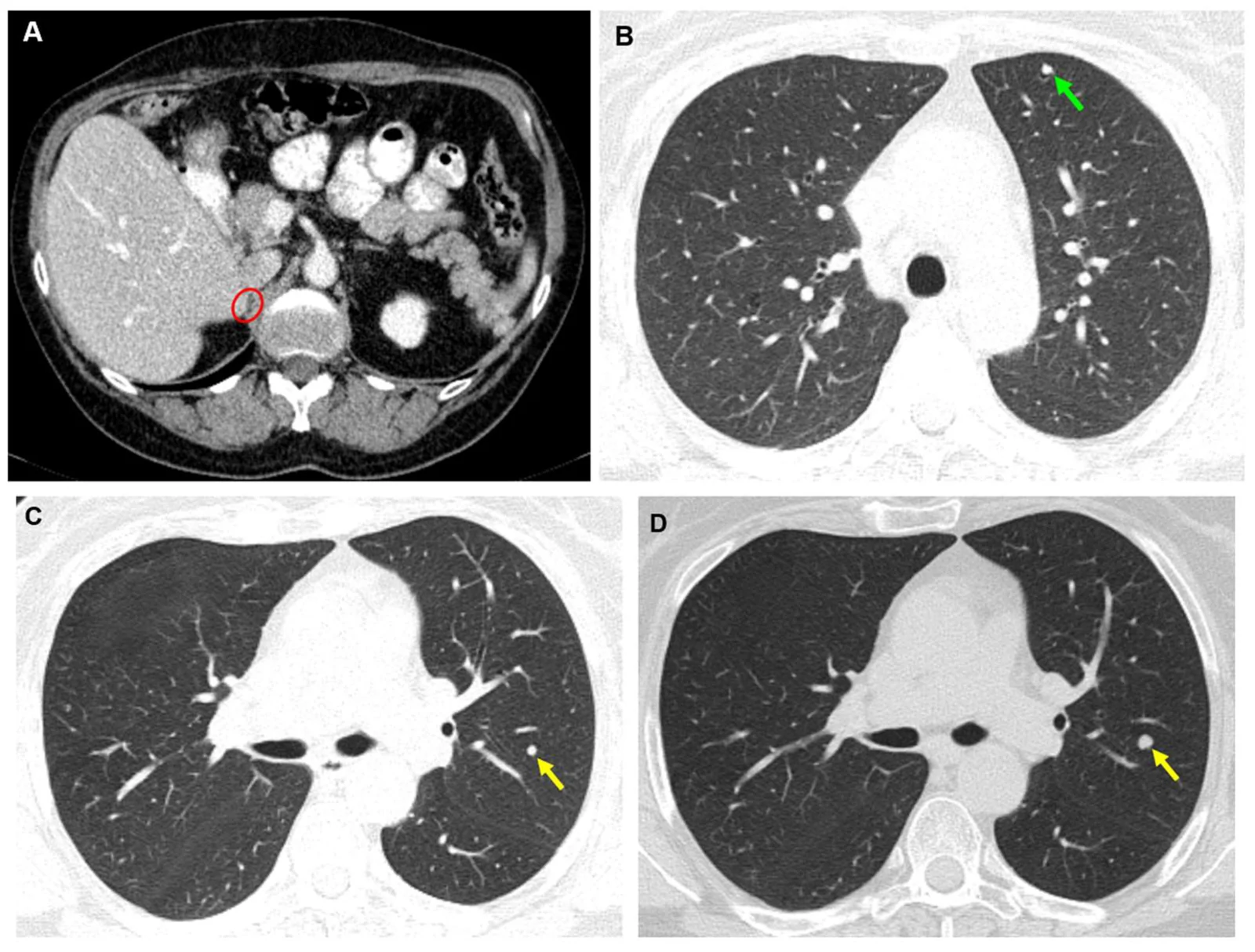

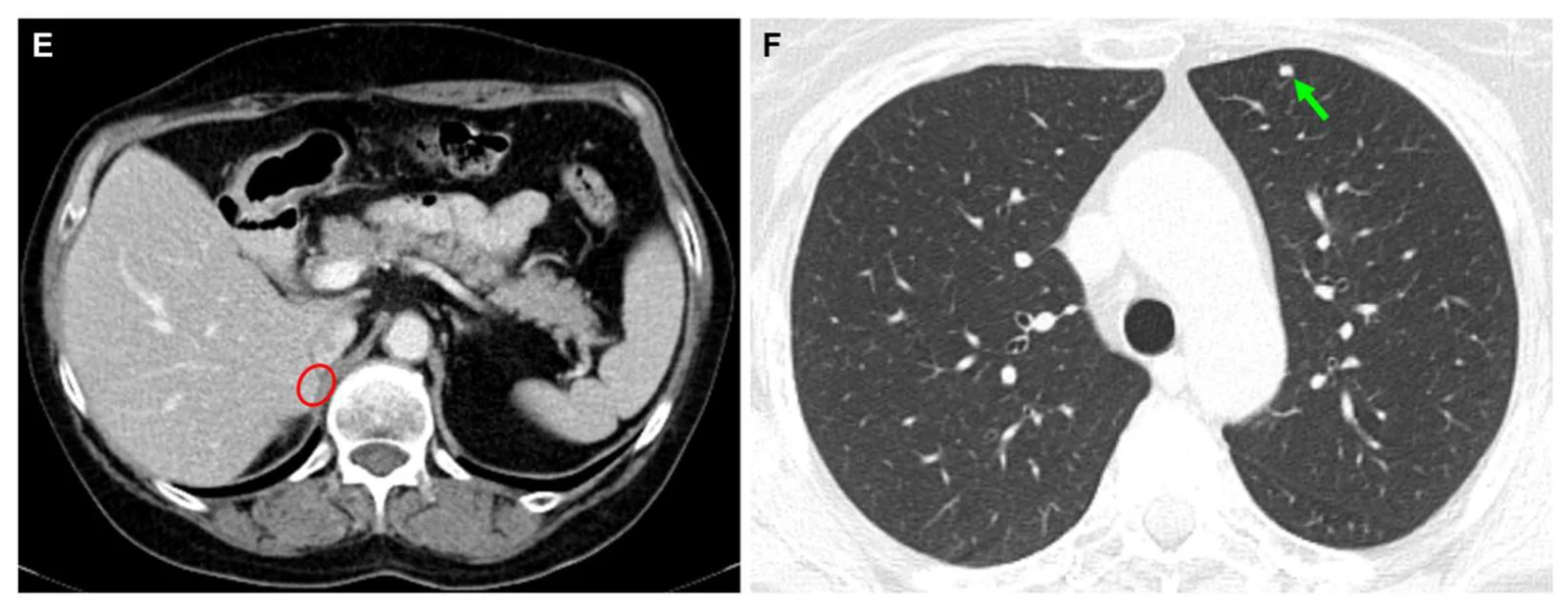
The patient underwent a 5-year protocol of mitotane (between 2012 and 2017), which she tolerated well. In the meantime, CT scans showed no local recurrence in the adrenal area and two stationary pulmonary micronodules. The adrenal failure was well controlled with 5 to 7.5 mg/day of oral prednisone and 0.1 mg/day of fludrocortisone. She developed primary hypothyroidism requiring daily oral levothyroxine therapy (50 µg/day and later 75 µg/day) and showed a tendency to ecchymoses and easy bruising (Figure S2). [CT scan in the year 2015 (**A**,**B**): (**A**) right adrenalectomy area with postsurgical fibrosis (red circle), no image of local tumoral recurrence (contrast-enhanced CT scan, axial plane, venous phase); (**B**) pulmonary nodule of 5.1 mm (green arrow) in the anterior segment of the left upper lobe (contrast-enhanced CT scan, axial plane, lung window); CT assessment in the year 2016 (**C**): (**C**) pulmonary nodule of 4.5 mm (yellow arrow) in the apico-posterior segment of the left upper lobe (contrast-enhanced CT scan, axial plane, lung window); CT scan in the year 2017 (**D**–**F**): (**D**) pulmonary micronodule of 6 mm (yellow arrow) in the apico-posterior segment of the left upper lobe (contrast-enhanced CT scan, axial plane, lung window); (**E**) right adrenalectomy area with postsurgical fibrosis (red circle), no image of local tumoral recurrence (contrast-enhanced CT scan, axial plane, venous phase); (**F**) pulmonary nodule of 5.1 mm (green arrow) in the anterior segment of the left upper lobe (contrast-enhanced CT scan, axial plane, lung window)].

**Figure 5:**
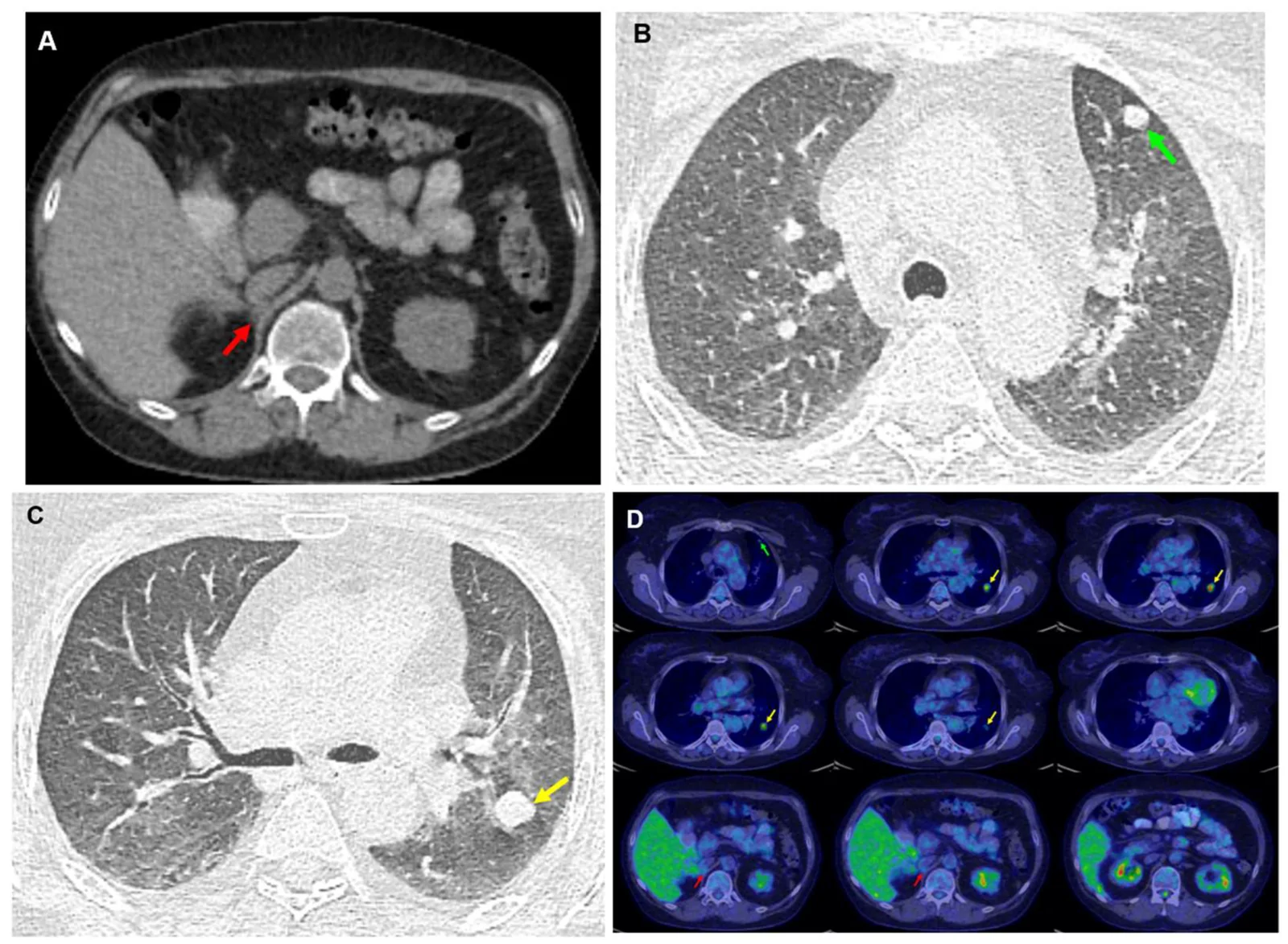
In accordance with the patient’s option for mitotane withdrawal, she remained under endocrine surveillance as an outpatient for another four years (between 2018 and 2020), while she felt well and continued all the mentioned replacement hormonal therapy for the adrenal and thyroid insufficiency [CT scan in the year 2018 (**A**–**C**): (**A**) right oval retrocaval mass (red arrow) of 8 by 7.5 by 11 mm indicating ACC recurrence (native CT scan, axial plane); (**B**) pulmonary nodule of 8 mm (green arrow) in the anterior segment of the left upper lobe (native CT scan, axial plane, lung window); (**C**) pulmonary nodule of 14 mm (yellow arrow) in the apico-posterior segment of the left upper lobe (native CT scan, axial plane, lung window); positron emission tomography (PET-CT, axial plane) in the year 2018 (**D**): pulmonary nodule of 8 mm (green arrow) in the anterior segment of the left upper lobe without focal metabolic activity; metabolically active pulmonary nodule of 14 mm (yellow arrow) in the apico-posterior segment of the left upper lobe; metabolically active right oval retrocaval mass of 8 by 7.5 by 11 mm (red arrow) suggestive of a local ACC recurrence].

**Figure 6:**
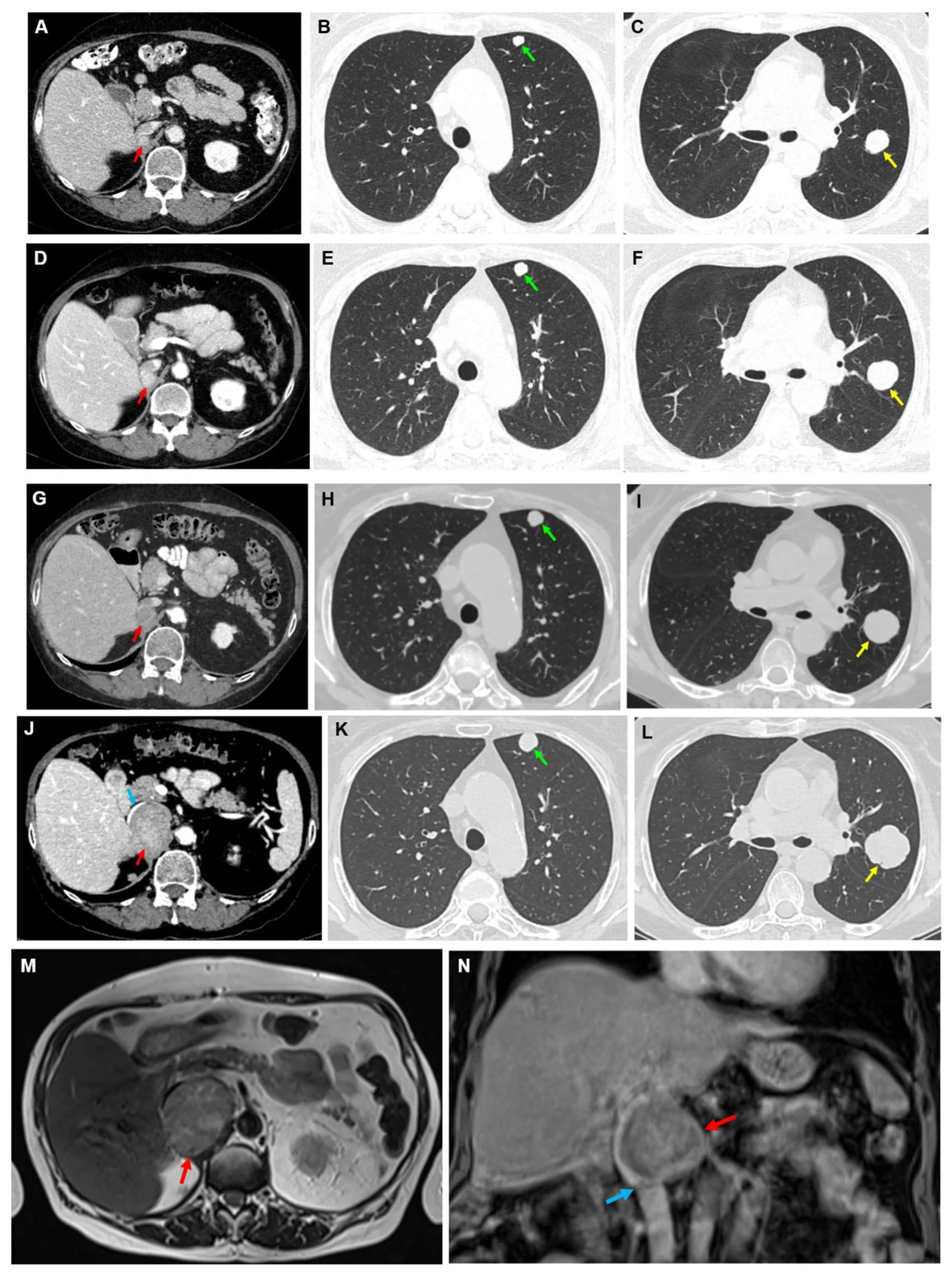
Further on, starting with late 2020, ACC showed a local recurrence, and both lung metastases progressively increased, as shown by CT scan. The patient only agreed to re-start oral mitotane as single-line therapy (which she continued between early 2021 and 2025) [CT scan in the year 2020 (**A**–**C**): (**A**) right oval, heterogeneous, retrocaval mass (red arrow) of 15.4 by 15.1 by 18 mm showing local ACC recurrence (contrast-enhanced CT scan, axial plane, venous phase); (**B**) pulmonary nodule of 8 mm (green arrow) in the anterior segment of the left upper lobe (contrast-enhanced CT scan, axial plane, lung window); (**C**) heterogeneous pulmonary nodule (yellow arrow) with lobulated margins of 19.5 by 20 by 17.4 mm in the apico-posterior segment of the left upper lobe (contrast-enhanced CT scan, axial plane, lung window); CT evaluation in the year 2021 (**D**–**F**): (**D**) right oval, heterogeneous, retrocaval mass (red arrow) of 14.4 by 16.7 by 18.3 mm showing ACC (contrast-enhanced CT scan, axial plane, venous phase); (**E**) pulmonary nodule of 13.5 by 11.2 by 13.7 mm (green arrow) in the anterior segment of the left upper lobe (contrast-enhanced CT scan, axial plane, lung window); (**F**) heterogeneous pulmonary nodule (yellow arrow) with lobulated margins of 23.8 by 22.3 by 28.4 mm in the apico-posterior segment of the left upper lobe (contrast-enhanced CT scan, axial plane, lung window); CT evaluation in the year 2022 (**G**–**I**): (**G**) right oval, heterogeneous, retrocaval mass (red arrow) of 18.2 by 20.3 by 21.1 mm (contrast-enhanced CT scan, axial plane, venous phase); (**H**) pulmonary nodule of 12.8 by 11.8 by 15.4 mm (green arrow) in the anterior segment of the left upper lobe (contrast-enhanced CT scan, axial plane, lung window); (**I**) heterogeneous pulmonary nodule (yellow arrow) with lobulated margins of 29.5 by 28.2 by 34.7 mm in the apico-posterior segment of the left upper lobe (contrast-enhanced CT scan, axial plane, lung window); CT scan in the year 2024 (**J**–**L**): (**J**) right heterogeneous, retrocaval mass (red arrow) of 51.8 by 61.3 by 49 mm, in close contact with the hepatic segment V; the inferior vena cava (blue arrow) was compressed by the adrenal mass (contrast-enhanced CT scan, axial plane, venous phase); (**K**) pulmonary nodule (green arrow) of 14.8 by 16.4 by 20 mm in the anterior segment of the left upper lobe, in close contact with the parietal pleura (contrast-enhanced CT scan, axial plane, lung window); (**L**) heterogeneous, spiculated pulmonary nodule (yellow arrow) with lobulated margins of 38.7 by 36 by 40 mm in the apico-posterior segment of the left upper lobe (contrast-enhanced CT scan, axial plane, lung window); abdominal magnetic resonance imaging (MRI) in the year 2024 (**M**,**N**): (**M**) right heterogeneous, retrocaval mass (red arrow) of 53 by 68 by 58 mm, in close contact with the hepatic segment V, indicating ACC (T2 TSE sequence, axial plane); (**N**) ACC (red arrow) recurrence (contrast-enhanced MRI scan, T1 vibe Dixon, coronal plane) and inferior vena cava (blue arrow) with anterior compression by the adrenal tumor (contrast-enhanced MRI scan, T1 vibe Dixon, coronal plane)].

**Figure 7:**
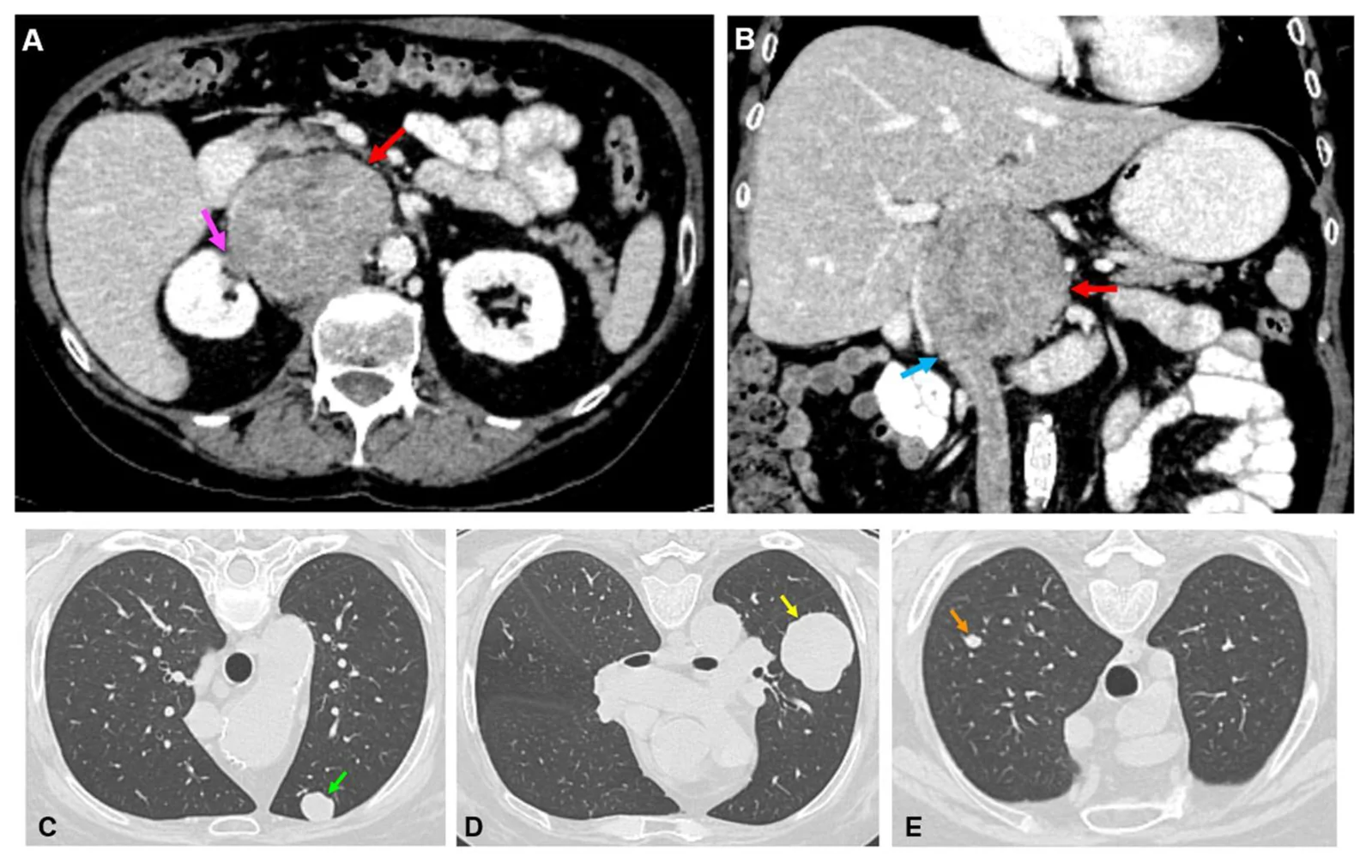

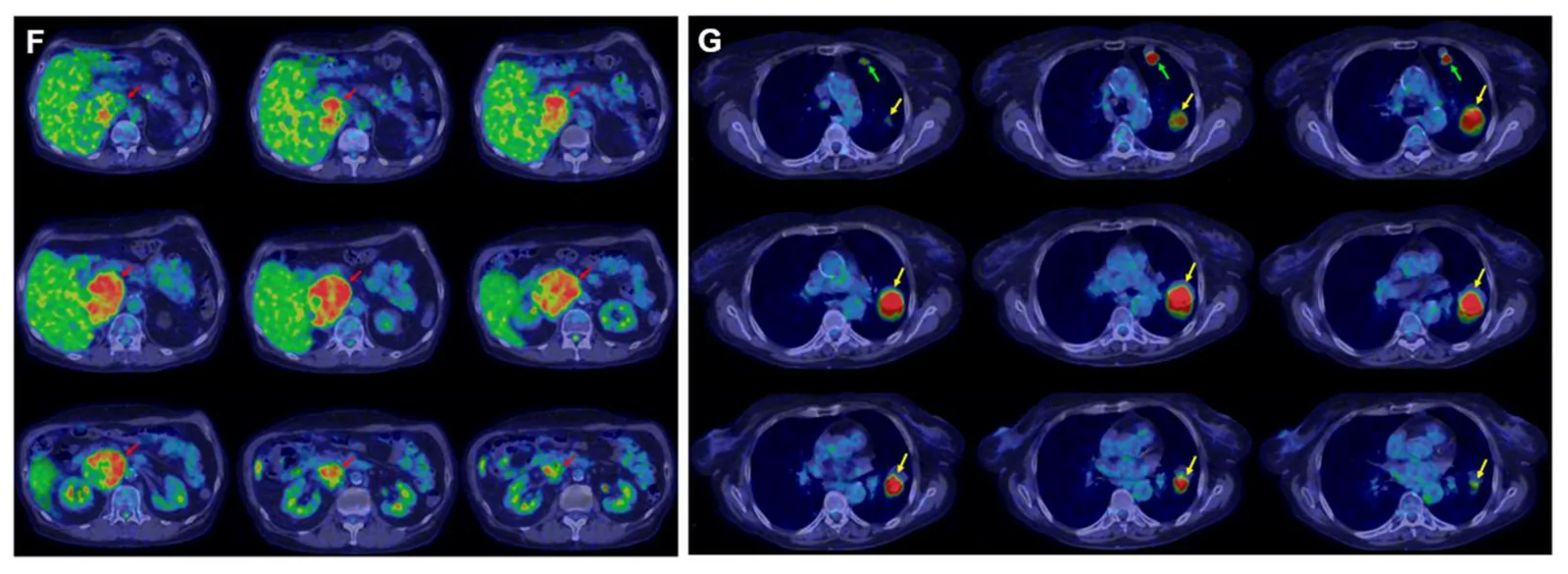
At the age of 65 (in the year 2025, approximately 15 years since the first presentation), a decision to perform a third adrenalectomy was made in accordance with the patient’s option. At this point, the adrenal mass increased to almost 12 cm on imaging evaluation [Contrast-enhanced CT scan, venous phase (**A**,**B**): (**A**) right heterogeneous, retrocaval mass (red arrow) of 70.4 by 82 by 110.8 mm and tumoral invasion (pink arrow) of the right kidney (axial plane); (**B**) ACC (red arrow) and inferior vena cava (blue arrow) compressed by the adrenal mass (coronal plane)]. The size of the lung metastases increased, and a potential novel micronodule was identified on the right [Contrast-enhanced CT scan, axial plane, lung window (**C**–**E**): (**C**) pulmonary nodule (green arrow) of 17.5 by 16.8 by 20 mm in the anterior segment of the left upper lobe, in close contact with the parietal pleura; (**D**) heterogeneous, spiculated pulmonary nodule (yellow arrow) with lobulated margins of 43.5 by 46.9 by 51.9 mm in the apico-posterior segment of the left upper lobe extending into the superior segment of the left lower lobe, in close contact with the left pulmonary hilum; (**E**) pulmonary nodule (orange arrow) of 7.3 mm in the apical segment of the right upper lobe]. PET-CT confirmed metabolically active sites at the right adrenal and both masses on the left lung [PET-CT scan, axial plane (**F**,**G**): (**F**) retrocaval, inhomogeneous, metabolically active mass (red arrow) of 74 by 60 by 80 mm; (**G**) metabolically active pulmonary nodule (green arrow) of 16 by 17 mm in the anterior segment of the left upper lobe, in close contact with the parietal pleura and metabolically active pulmonary nodule (yellow arrow) of 40 by 42 by 45 mm in the apico-posterior segment of the left upper lobe].

**Figure 8:**
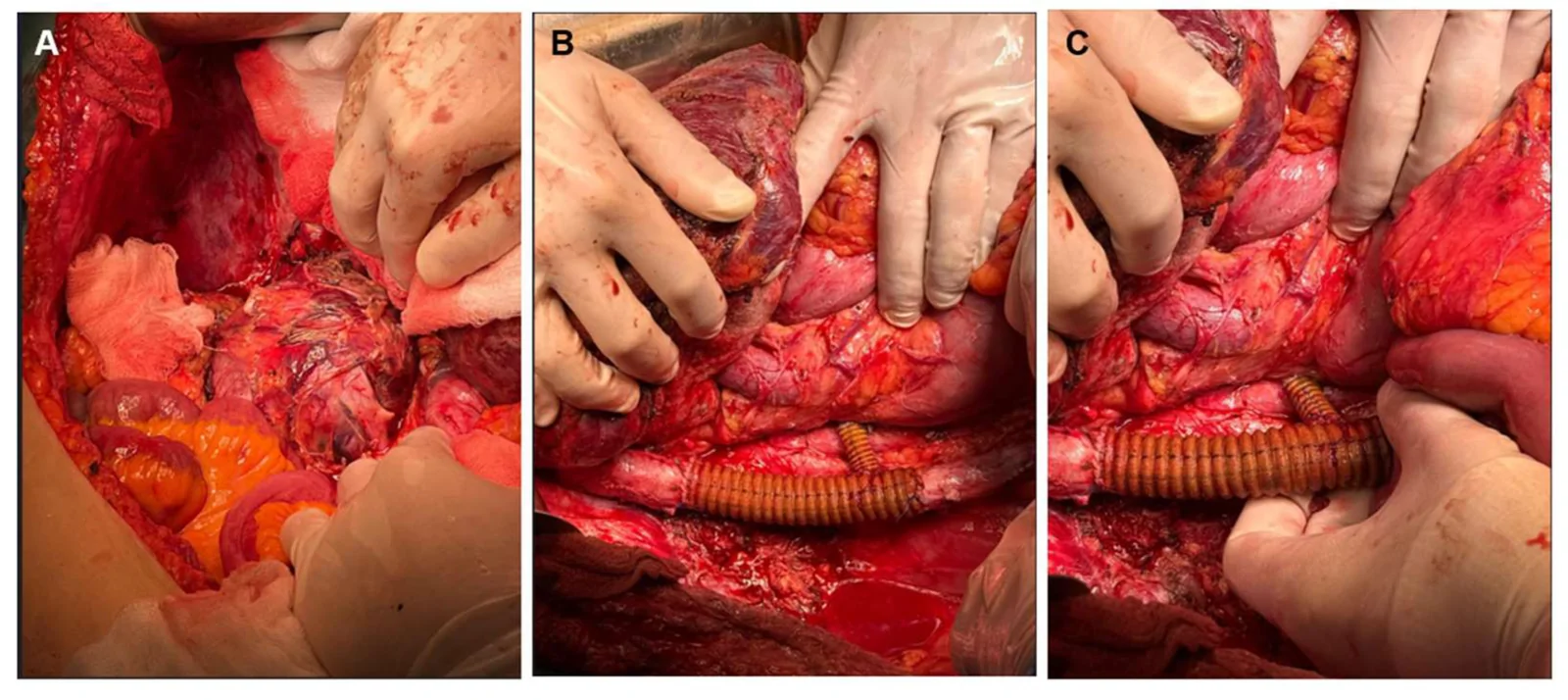
Radical, multi-visceral, en bloc resection was done, including: the right adrenal tumor, the right kidney, three liver segments (segment 1 and atypical segments 6 and 7), the retro-hepatic inferior vena cava, and (partial) left renal vein. The reconstruction of the inferior vena cava required a Dacron graft of 12 cm in addition to a synthetic graft, which was placed between the left renal vein and the first graft due to the small length of the renal vein. [(**A**) Intraoperative capture; (**B**,**C**) graft-graft anastomosis]. The patient slowly recovered and experienced a partial wound dehiscence. During surgery and the following weeks, she presented with several episodes of acute adrenal insufficiency, which required even higher doses of intravenous hydrocortisone, despite receiving continued prompt replacement. A peak adrenocorticotropic hormone level (ACTH) of 369 (normal range: from 7.2 to 63.3) pg/mL was reached at one point. Renal function remained stable, with a maximum creatinine of 1.01 (normal range: between 0.6 and 1.25) mg/dL and urea of 58 (normal range: between 15 and 36) mg/dL. During this time, she continued levothyroxine replacement (75 µg/dL) (Figure S3).

**Figure 9:**
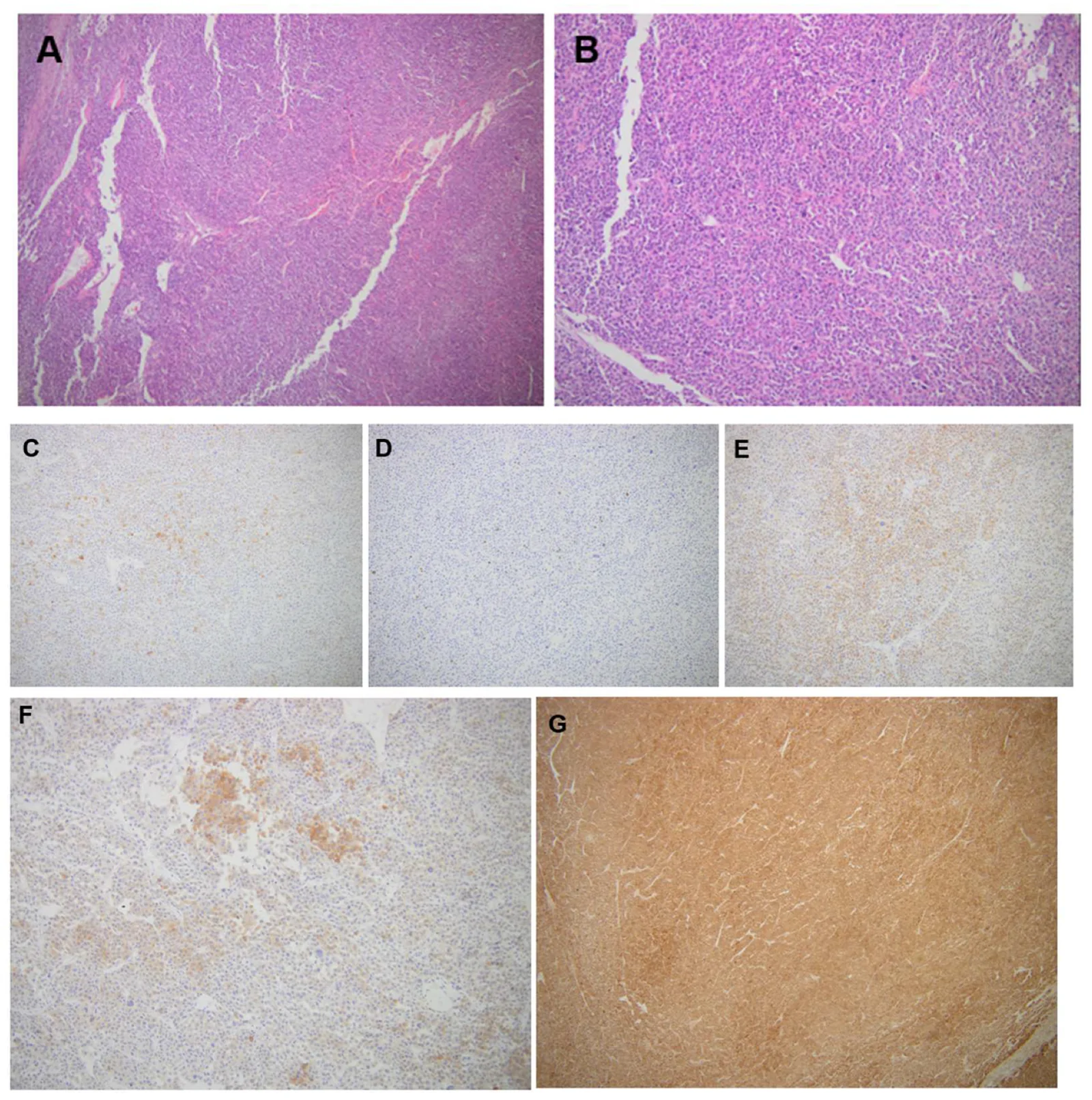
Postoperative pathological report confirmed a low-grade ACC with the same histological and immunohistochemical features as those found after previous adrenalectomies. [Histological analysis; hematoxylin-eosin (**A**,**B**) showing intense cellularity with oval and polygonal cells, increased pleomorphism, eosinophilic cytoplasm, round or oval nuclei, frequent mitoses: (**A**) 4X; (**B**) 10X; immunohistochemistry report (**C**–**G**): (**C**) inhibin positive in tumor cells (10X); (**D**) Ki67 of 15% (10X); (**E**) melan A positive in tumor cells (10X); (**F**) synapto positive in tumor cells (10X); (**G**) calretin positive in tumor cells (4X)].

**Figure 10:**
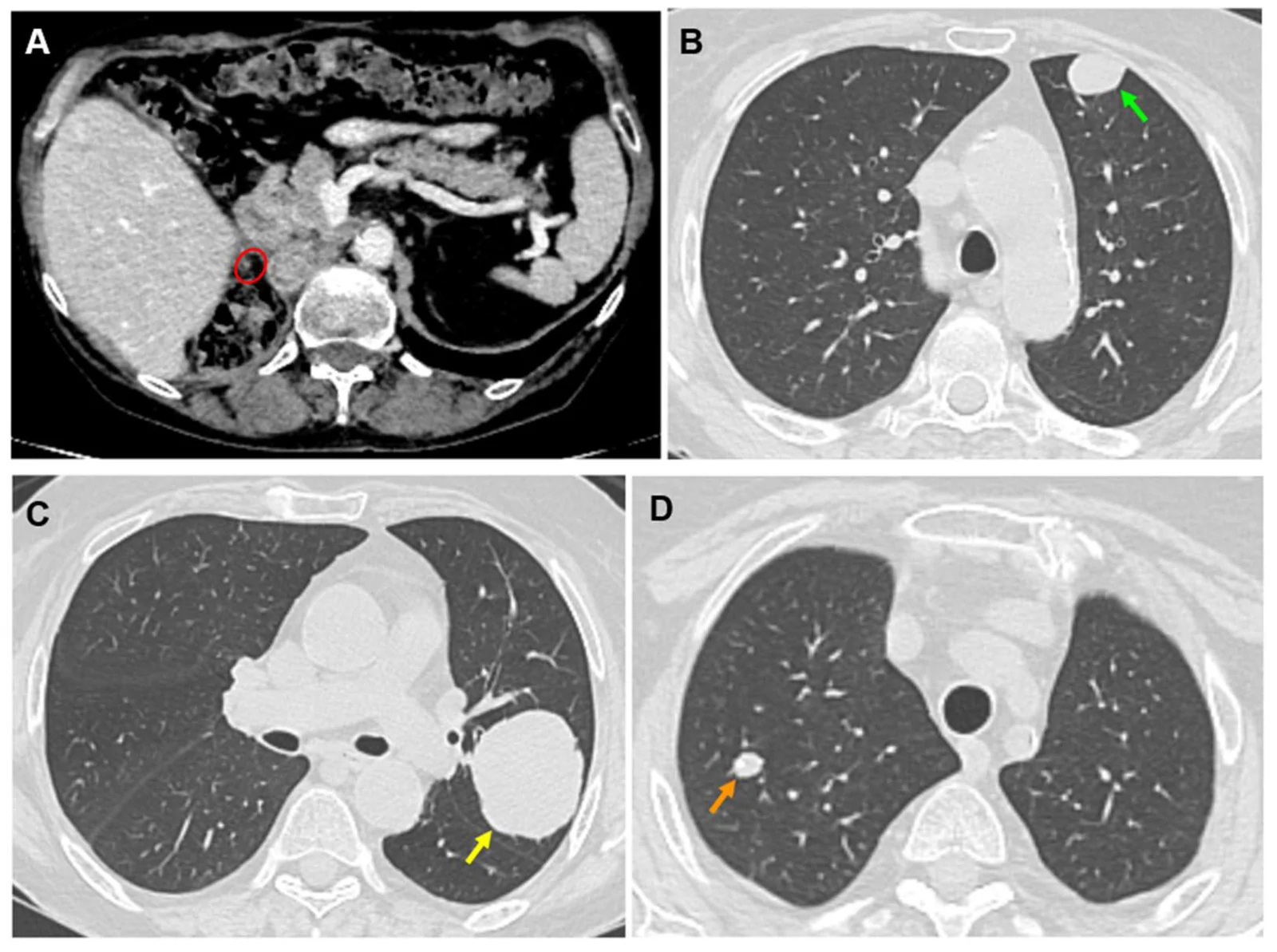
The patient re-started daily mitotane with progressive dose increase starting with month 3 post-surgery. Thoracic surgery for the lung metastases was considered high risk and was not performed. She still refused any other type of adjuvant treatment (chemotherapy, immunotherapy), while she has completely recovered and felt well during the 9 months following the third adrenal intervention. Further close surveillance is mandatory. [CT scan at 4 months since the third adrenalectomy (**A**–**D**): (**A**) resection area (red circle) of ACC with no image of remnants (contrast-enhanced CT scan, axial plane, venous phase); (**B**) pulmonary nodule (green arrow) of 20.8 by 21.7 by 29 mm in the anterior segment of the left upper lobe, in close contact with the parietal pleura (contrast-enhanced CT scan, axial plane, lung window); (**C**) heterogeneous, spiculated pulmonary nodule (yellow arrow) with lobulated margins of 44 by 51 by 59 mm in the apico-posterior segment of the left upper lobe extending into the superior segment of the left lower lobe, in close contact with the left pulmonary hilum (contrast-enhanced CT scan, axial plane, lung window); (**D**) pulmonary nodule (orange arrow) of 8.4 mm in the apical segment of the right upper lobe (contrast-enhanced CT scan, axial plane, lung window)]. To date, ACC represents a very rare and aggressive malignancy (an incidence of 1 to 2 cases per million and an overall 5 year-survival of 15%), and less than 15-20% of cases involve a single metastatic disease [1,2,3,4,5]. This was a real-life setting with a personalized approach that mandatorily took into consideration the patient’s option to refuse chemotherapy, immunotherapy, or radiotherapy at any point in life and delay surgery at some moments, as shown above (including during the COVID-19 pandemic and early post-pandemic years). Despite this decision-making, survival for more than 16 years since the first identification of the tumor qualifies the case as one of the rarest of its kind [1,2,3,4]. Notably, triple adrenal surgery over two decades also indicated exceptional management, in addition to the fact that the third procedure was a multi-organ, en bloc, radical resection (with cave-cave shunt using synthetic grafts), which is limited to a few previously published cases worldwide (and none in our country) [5,6,7]. This procedure can only be performed by highly skilled surgical teams. To the best of our knowledge, this is the first case in which this type of procedure was done on a patient who survived with ACC for 15 years after two prior adrenalectomies. Notably, the adrenalectomy (if feasible) represents the most important contributing factor to the overall prognosis (other than the tumor’s features and clinical picture/comorbidities of one patient) in association with the significant role of the adjuvant adrenolytic agent mitotane [8,9,10]. No other genetic/molecular testing was agreed to by the patient. Of note, the consistent pathological/histological profile of this low-grade ACC, which was confirmed over the years in both adrenal and lung sites, might explain the prolonged survival in this particular instance. Perhaps this prolonged survival (or better prognosis) could be explained by molecular/genetic research that the patient refused to undergo, but since no such research was undertaken, this should be strongly underlined as a study limitation. Yet, a sub-category of ACCs potentially has an overall good prognosis when compared with the prognosis suggested by most of the evidence we have so far [11,12,13,14], and potentially the current characterization might miss some clues under these specific circumstances. Hence, awareness remains the key operating factor.

## Data Availability

All available data are in the article.

## Supplementary Materials

The following supporting information can be downloaded at: https://www.mdpi.com/article/10.3390/diagnostics16183006/s1, Figure S1: Flowchart diagram of the mitotane therapy (from 2012 until 2026); Figure S2: Ecchymoses under mitotane therapy (particularly at hands and forearms areas), either spontaneous or at mild trauma or after blood withdrawn punction; Figure S3: Thyroid ultrasound amid mitotane-induced primary hypothyroidism: hypoechoic, inhomogeneous pattern with right lobe of 14 by 11 by 37 mm and left lobe of 16 by 11 by 31 mm (transverse plane).
