# Supplementary material for: Adrenocortical Carcinoma: An Exceptional Survival Span Across a Tailored Management
Source: Diagnostics (Basel). 2026 Sep 17;16(18):3006. doi: 10.3390/diagnostics16183006 (PMC13605629; doi:10.3390/diagnostics16183006)

Supplementary Materials/Appendix

Figure S1. Flowchart diagram of the mitotane therapy (from 2012 until 2026).

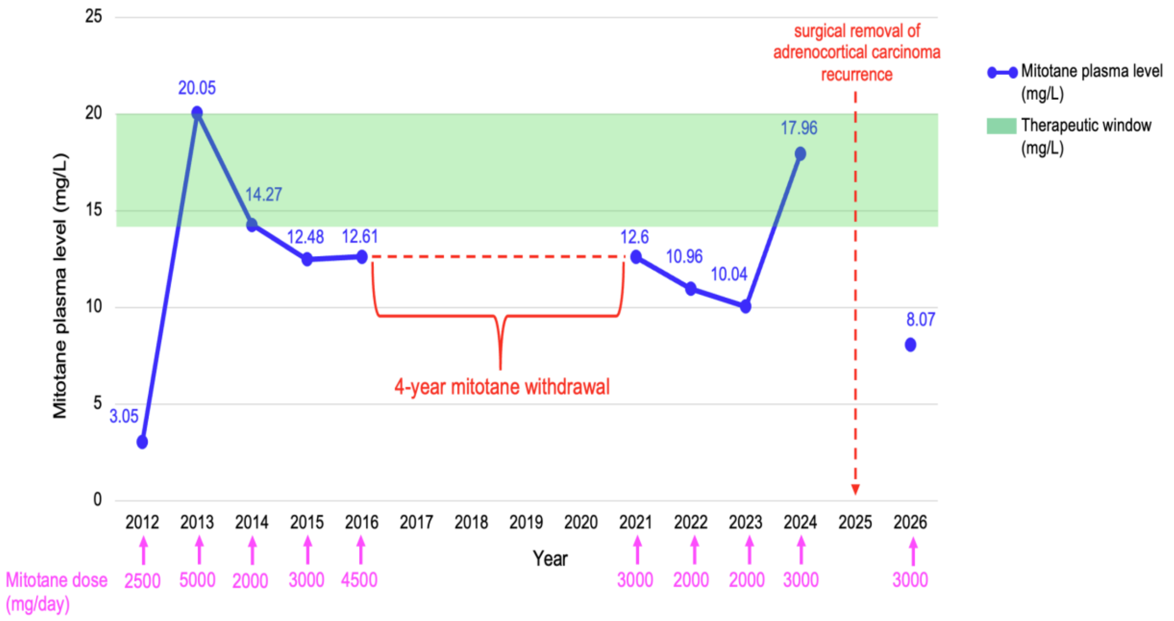

Figure S2. Ecchymoses under mitotane therapy (particularly at hands and forearms areas), either spontaneous or at mild trauma or after blood withdrawn punction.

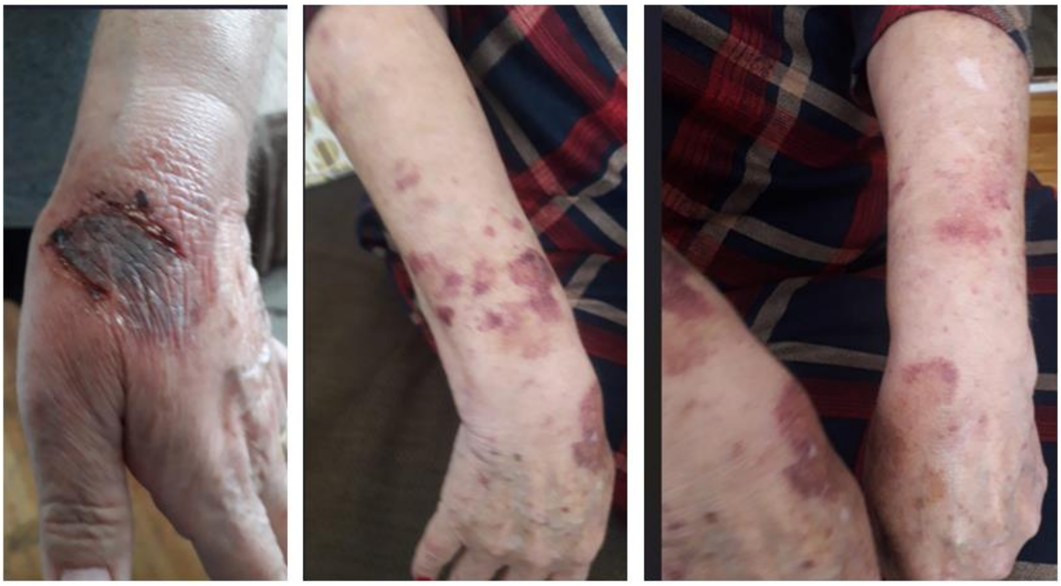

Figure S3. Thyroid ultrasound amid mitotane-induced primary hypothyroidism: hypoechoic, inhomogeneous pattern with right lobe of 14 by 11 by 37 mm and left lobe of 16 by 11 by 31 mm (transverse plane).

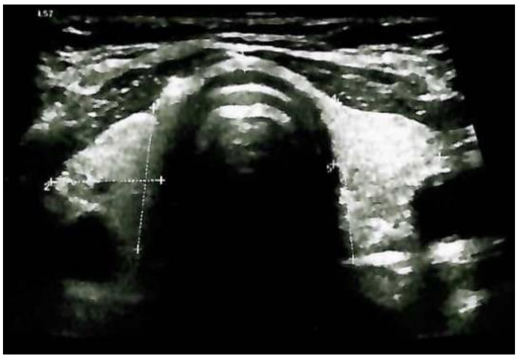

Supplement: Supplementary file 1 [file diagnostics-16-03006-s001.zip › diagnostics-4519261-supplementary.pdf]
